# B. Bradshaw's Dictionary of Bathing Places and Climatic Health Resorts

**Published:** 1902-09

**Authors:** 


**B. Bradshaw's Dictionary of Bathing Places and Climatic Health Resorts.** Pp. xl., 372. London: Kegan Paul, Trench, Trübner & Co. 1902.

The invalid going to a foreign Spa will do well to study this volume before he makes his choice. It gives him much useful instruction, and every effort has been made to eliminate errors.

The volume contains all necessary information, except the invaluable and most interesting time table bearing also the name of Bradshaw.

